# Task-related modulation of anterior theta and posterior alpha EEG reflects top-down preparation

**DOI:** 10.1186/1471-2202-11-79

**Published:** 2010-06-28

**Authors:** Byoung-Kyong Min, Hae-Jeong Park

**Affiliations:** 1 Department of Radiology, Brigham and Women's Hospital, Harvard Medical School, Boston, USA; 2 Brain Korea 21 Project for Medical Science, Department of Radiology, Nuclear Medicine and Research Institute of Radiological Science, Yonsei University College of Medicine, Seoul, Korea

## Abstract

**Background:**

Prestimulus EEG alpha activity in humans has been considered to reflect ongoing top-down preparation for the performance of subsequent tasks. Since theta oscillations may be related to poststimulus top-down processing, we investigated whether prestimulus EEG theta activity also reflects top-down cognitive preparation for a stimulus.

**Results:**

We recorded EEG data from 15 healthy controls performing a color and shape discrimination task, and used the wavelet transformation to investigate the time course and power of oscillatory activity in the signals. We observed a relationship between both anterior theta and posterior alpha power in the prestimulus period and the type of subsequent task.

**Conclusions:**

Since task-differences were reflected in both theta and alpha activities prior to stimulus onset, both prestimulus theta (particularly around the anterior region) and prestimulus alpha (particularly around the posterior region) activities may reflect prestimulus top-down preparation for the performance of subsequent tasks.

## Background

When people identify an object, they have to match what they sense against their knowledge. In general, people accomplish recognition by a combination of two information processing pathways: *top-down *and *bottom-up*. Bottom-up processing (i.e. sensation) occurs when the sensory input information induces perceptual representation, whereas top-down processing (i.e. identification) occurs when the perceptual representation is influenced by some higher mental function such as previous knowledge, motivation, or expectation.

To facilitate perceptual identification, one may use subjective expectations of the stimulus to come. Accordingly, a cognitive intention (e.g. expectation, mental readiness, active redirection of attention), embedded in a top-down process, may precede an event or stimulus. Bottom-up sensory processing is then guided by such top-down processing as a specific reallocation of attention relevant to the type of stimulus to follow or task to be performed. In this way, top-down intentional processing can increase the speed and efficiency of perceptual identification.

In this regard, the influence of prestimulus mental activity on the subsequent poststimulus responses (or task-performances) is worth investigating. Indeed, some studies showed the relationship between the prestimulus alpha activity and the poststimulus event-related potential (ERP) components. Brandt's and Barry's groups have reported that greater prestimulus alpha amplitude led to larger ERP amplitudes [1-5], whereas Başar's group has found an inverse relation between prestimulus root-mean-square (RMS) alpha power and subsequent ERP amplitudes [6-9]. As it is known, ERPs are based on the recording of brain electrical potentials synchronized with the presentation of external sensory stimuli (so-called 'exogenous') as well as the occurrence of internal cognitive events (so-called 'endogenous') [10-12]. Likewise, there is still a fundamental debate on whether ERP components are generated from the ongoing EEG activity (e.g. alpha) by means of phase-reset [13-19]. In general, ERP components are influenced by bottom-up (sensory or physical) factors and also reflect top-down (cognitive) processing. However, since those studies show inconsistent observations, the relationship between the prestimulus alpha power and the poststimulus ERP components has been still controversial.

Meanwhile, using a color-shape discrimination task, Min and Herrmann [20] recently found that the prestimulus level of alpha activity differed significantly with the type of poststimulus task, and interpreted this to mean that prestimulus alpha activity reflects top-down preparation for the stimulus. Accordingly, the prestimulus mental state, typically regarded as a resting state and thus a baseline for poststimulus activity, may in fact be a task-relevant top-down control stage. Some previous studies consistently support this conception [21,22]. For instance, Ergenoglu et al. [21] observed that alpha activity modulates visual detection performance in humans.

Besides, Klimesch et al. [23] postulated that alpha synchronization might reflect a top-down function in inhibiting task-irrelevant information, since the event-related synchronization in the alpha band can be noticeably observed during task-performance either under such conditions where subjects have to withhold task-relevant information or over the brain regions that are task-irrelevant [24-30]. Therefore, by means of two kinds of discrimination tasks requiring inhibition of concurrent task-irrelevant feature processing for improving task-performance, here we would like to test a putative relationship between prestimulus EEG dynamics and poststimulus responses of task-performance from the viewpoint of top-down inhibitory preparation to the task-irrelevant feature of subsequently presented stimuli. Moreover, Mordkoff and Yantis [31] reported that coactivation occurs when target attributes from two separable dimensions are simultaneously present, but not when target attributes come from the same dimension. They argued dividing attention between color and shape as evidence of coactivation. To induce such coactivation, we employed target attributes from two dimensions (color and shape). This required subjects to inhibit the task-irrelevant feature to improve performance. Presumably, different task-difficulties across the color and shape tasks may induce differences in top-down preparation such as prestimulus task-performance strategy.

In addition, theta activity has received attention as a possible electrophysiological correlate of top-down processing. It has been reported that spontaneous EEG theta activity influences the amplitude of frontal visual evoked potentials [32], and that Freunberger et al. [33] suggested that phase-locked theta activity reflects top-down regulation in information memory systems. Sauseng et al. [34] also suggested that theta activity may represent top-down processing, and gamma activity bottom-up processing, based on observations of enhanced phase-synchrony between theta and gamma bands for an attended visual target. Studies consistently suggest that slow oscillations (e.g. theta and alpha) are related to function in large, distributed networks [35,36], whereas high frequency oscillations (e.g. gamma) are associated with neural processes in more local networks [36]. These studies support the conclusion that integration between top-down processes depends critically on slow oscillations such as alpha and theta.

Most studies on theta oscillations as a measure of top-down processes have focused on event-related (or poststimulus) modulations, and thus neglect prestimulus theta activity as ongoing top-down preparation for the performance of subsequent tasks. Note that a prestimulus mental state differs from a spontaneous mental state (i.e. a resting state with no tasks) in that mental activity in a prestimulus period would be influenced by the attributes of subsequent tasks or events. The prestimulus mental state may therefore present an appropriate target to explore differences in top-down regulation in advance of different task performances. We hypothesized that if theta activity reflects top-down processing, it must be largely controlled by ongoing top-down inhibitory processing, particularly during a prestimulus mental state almost entirely lacking in physical attributes of upcoming stimuli (i.e. bottom-up processes). In the present study we therefore investigated whether prestimulus theta activity represents a state of cognitive readiness, as prestimulus alpha activity does [20].

## Results

Pairs of colored figures randomly drawn from a set of red or green circles or squares were presented, and participants were requested to respond with the index finger of one hand if the target feature of the task ('color' in the color task and 'shape' in the shape task) was the same and to respond with the other hand if it was not. As a result, the shape task yielded significantly longer reaction times than the color task (F(1,14) = 5.2, *p *< 0.05; color task: 486 ms, shape task: 528 ms). For the accuracy of the task-performance, we also found a strong 'task' effect (F(1,14) = 15.0, *p *< 0.005), indicating that the color task performance showed significantly higher accuracy than the shape task performance (color task: 96.6%, shape task: 74.2%).

**Figure 1:**
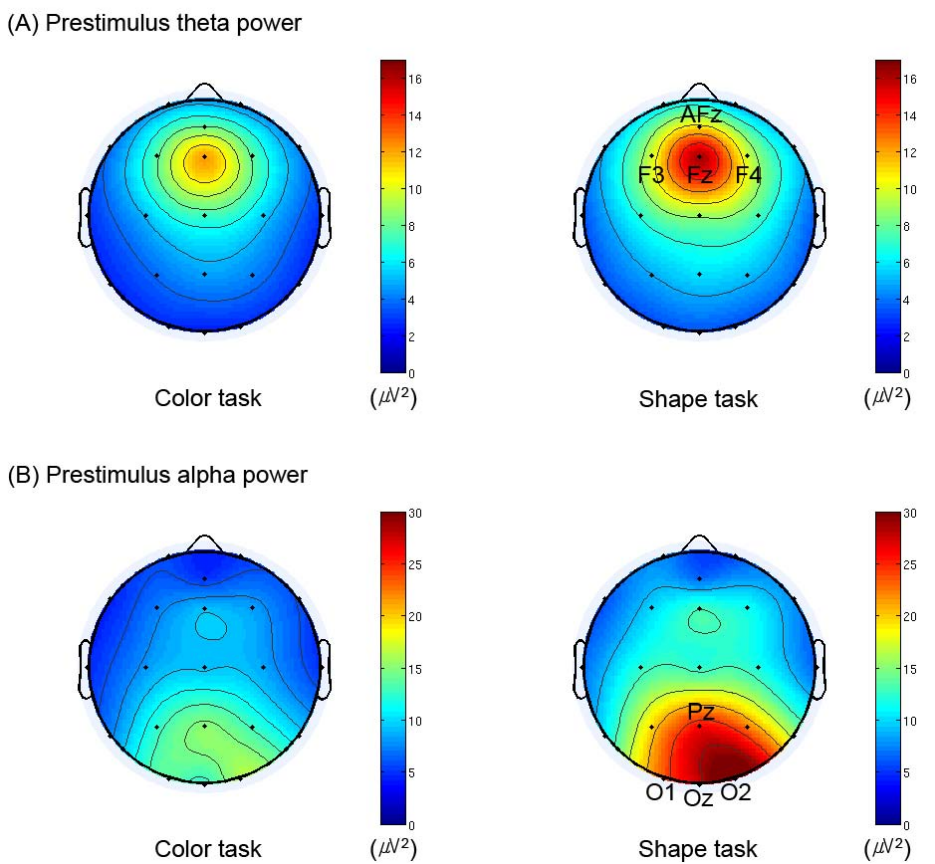
**Grand-averaged topographies of (A) prestimulus theta and (B) prestimulus alpha power for both tasks**. These topographical distributions were computed by averaging the mean power at subjects' individual alpha and theta frequencies over the time window from 300 to 50 ms prestimulus. Within this time window, individual alpha and theta frequencies were obtained from the frequencies showing maximal power of each task in alpha band on the electrode Oz and in theta band on the electrode Fz, respectively. Note the differences in prestimulus alpha power around the posterior region (including the electrodes Pz, O1, Oz and O2), and those of prestimulus theta power around the anterior region (including the electrodes AFz, F3, Fz and F4) between the two tasks. All views are from the vertex, and the upside is nasal. Color bars indicate scales of the power (μV^2^) of oscillatory activity.

**Figure 2:**
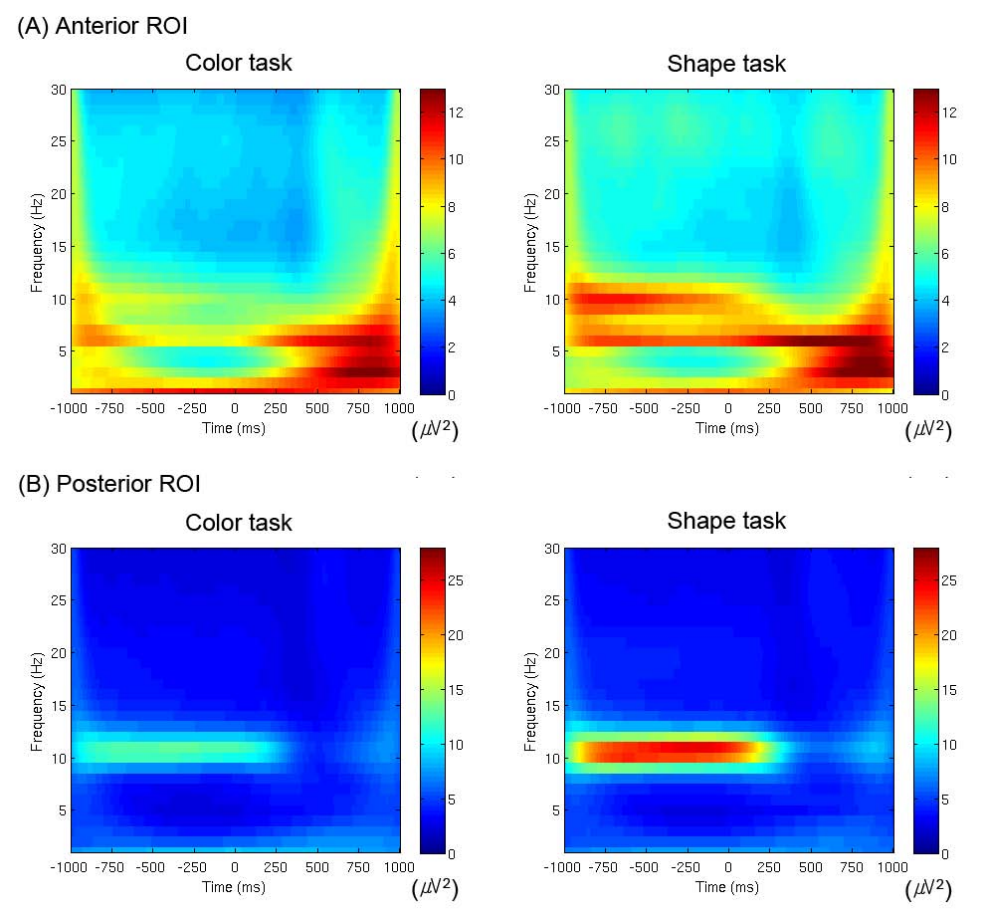
**Grand-averaged time-frequency representations of total power of oscillatory activity (1-30 Hz) on (A) the anterior ROI and (B) the posterior ROI for both tasks**. Stimuli were presented from 0 to 500 ms. Color bars indicate scales of the power (μV^2^). Note the alpha activity (about 10 Hz) on the posterior ROI (averaged across the electrodes Pz, O1, Oz and O2) and the theta activity (about 6 Hz) on the anterior ROI (averaged across the electrodes AFz, F3, Fz and F4), and compare the differences between the two tasks, particularly before stimulus onset.

As shown in Figs. 1 and 2, we observed the significant main effect of 'task' (F(1,14) = 5.1, *p *< 0.05) with higher prestimulus total alpha power for the shape task (color task: 10.9 μV^2^, shape task: 18.7 μV^2^). We also found that the shape task was preceded by significantly higher prestimulus total theta power than the color task (F(1,14) = 5.0, *p *< 0.05; color task: 6.0 μV^2^, shape task: 7.7 μV^2^). In addition, we observed that the anterior ROI showed significantly higher prestimulus total theta power than the posterior ROI (F(1,14) = 10.0, *p *< 0.01; anterior ROI: 10.2 μV^2^, posterior ROI: 3.5 μV^2^; cf. Figs. 1 and 2).

Compared to the posterior region, the anterior region showed significantly higher poststimulus total power of both alpha (F(1,14) = 4.8, *p *< 0.05; anterior ROI: 12.9 μV^2^, posterior ROI: 10.4 μV^2^) and theta activity (F(1,14) = 5,0, *p *< 0.05; anterior ROI: 19.1 μV^2^, posterior ROI: 14.6 μV^2^). In addition, the shape task showed significantly lower poststimulus alpha power than the color task (F(1,14) = 7.9, *p *< 0.05; color task: 12.3 μV^2^, shape task: 11.0 μV^2^). However, the 'task' factor did not have a significant effect on the poststimulus theta power.

The topographical distributions for both alpha and theta power between the two tasks are presented in Fig. 1. As shown, the prestimulus anterior theta and posterior alpha power were more intense in the shape task than in the color task. Fig. 2 shows grand-averaged time-frequency representations of power for total activity on the anterior and posterior ROIs. Note that the theta power had its maximum after the stimulation, whereas the alpha power showed dominance and higher power before stimulation.

## Discussion

We observed more intense anterior theta and posterior alpha power in the prestimulus phase of the shape task than of the color task. One might possibly suspect that the effects of prestimulus activity come from prolonged poststimulus responses. To validate the present effects as prestimulus effects, we performed an additional experiment with longer inter-stimulus intervals (ISIs; 2500-3500 ms as compared with the present ISIs of 1500-2500 ms). Eventually, the same results were consistently obtained although we replicated the experiment with longer ISIs (Min et al., unpublished observations). Therefore, one can exclude a possibility that prestimulus effects are confounded by tailing of poststimulus responses. Moreover, since the prestimulus oscillatory activity was measured sufficiently far from the mean latency for button-responses (about 507 ms poststimulus; subsequently the response-prestimulus time interval ranged approximately from 2200 to 3200 ms), our observations in the prestimulus period are also out of the possible confounding ranges by response-related brain activity.

Based on behavioral results, the color feature may be more salient, and thus harder to ignore than the shape feature, in the successful performance of the shape task [20]. To suppress such salient task-irrelevant information as color, the shape task may demand more attention-readiness. The present study extends previous findings that prestimulus alpha activity reflects 'cognitive readiness' for an upcoming task [20,37-40], with evidence that prestimulus theta activity may have similar functional significance. It is noteworthy that theta power showed its greatest intensity in the medial frontal region (see topographies, cf. Fig. 1A). Based on previous studies [41,42], frontal midline theta activity seems to principally reflect alternative activation of the anterior cingulate cortex (ACC) and prefrontal cortex. Wang et al. [43] suggested that theta activity from the ACC might reflect active inhibition. They observed transient phase locking of task-relevant theta activity in relation to active inhibition in superficial cingulated layers. Yordanova et al. [44] also proposed that theta oscillations particularly in relation to movement execution are functionally associated with error monitoring, of which the ACC is mainly in charge [45]. In line with these findings, Hanslmayr et al. [46] reported that theta activity in the ACC increased linearly with increasing interference during the stroop task, and concluded that sustained phase coupling between the ACC and the prefrontal cortex most likely reflects the engagement of cognitive control mechanisms. These findings support a significant relationship between frontal midline theta activity and inhibitory control of task-irrelevant processing. As task difficulty increased in the present study, the demand for inhibitory control in cognitive preparation most likely increased, and resulted in significant differences in theta power levels between the two tasks. This interpretation is in accordance with the assumption by Klimesch et al. [26] that theta power reflects (at least in part) task difficulty and cognitive load.

A recent EEG-fMRI study [47] also showed enhancement of frontal theta and occipital alpha power during a modified Sternberg working memory task. While we observed modulations of anterior theta and posterior alpha power in the prestimulus period, they reported similar phenomena in the 'maintenance' phase during a working memory task. Our present observations in the prestimulus period and their observations in a working memory retention phase are all consistent with modulation of alpha and theta frequency bands depending on the current cognitive load. Understandably, the mental load increases with the attention a task demands, and induces greater alertness, which can help either strengthened inhibition of task-irrelevant processing or enhanced attention to task-relevant processing. These different experimental phases (i.e. 'prestimulus'; versus 'retention' period) may in fact represent analogous mental states, and thus share in part the same neural networks.

To integrate top-down influences into bottom-up information requires long-range communications, which slow oscillations such as alpha and theta activities might achieve [35,36,48]. The interplay between alpha and theta oscillations may, for example, reflect the transfer of information between memory systems [49], and the central executive functions of working memory may be reflected in the fronto-parietal coherence in alpha and theta bands [35,50]. Since the functional coupling between anterior and posterior brain regions may be essential to accomplish top-down control [51], the interactions between anterior theta and posterior alpha activities and top-down regulation processing must be explored in future research.

## Conclusions

The present study extends our previous findings that prestimulus alpha activity reflects ongoing top-down preparation for the performance of upcoming tasks [20,40], by evidence that prestimulus theta activity may have similar functional significance. Based on both behavioral and electrophysiological results, the difficult task (shape task), which requires more inhibition of the competing color feature, was preceded by significantly higher alpha and theta power as compared to the easy task (color task). Since such task-differences were reflected in both alpha and theta activities prior to stimulus onset, both prestimulus alpha and prestimulus theta activities may reflect top-down cognitive processing in preparation for the performance of subsequent tasks.

## Methods

### Subjects

Fifteen normal healthy volunteers (9 females, mean age 23; range 18-29 years) participated in this study, in accordance with the ethics guidelines at the Institutional Review Board of Yonsei University and the Declaration of Helsinki (World Medical Association: Ethical Principles for Medical Research Involving Human Subjects, 1964). Subjects gave informed consent prior to the start of the experiment. All had normal or corrected-to-normal vision, and none was color-blind, as determined by the *Ishihara *color test. None had a personal or family history of psychiatric disorders.

### Stimuli and procedure

**Figure 3:**
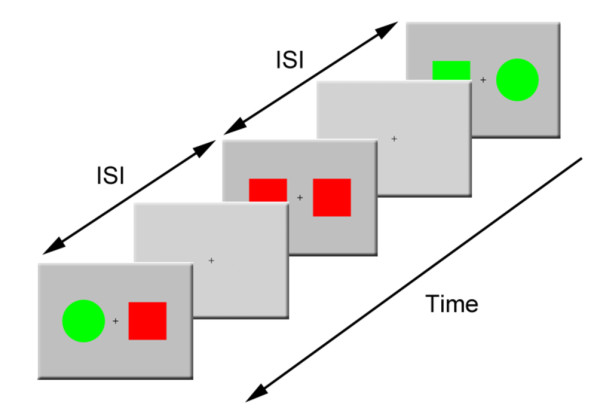
**A task flow diagram of sample stimuli and the ISIs**. Two stimuli randomly drawn from a set of red or green circles or squares were presented bilaterally against a light-gray background on a computer monitor. Stimulus presentation was followed by a fixation cross presented during every ISI. The ISIs ranged randomly from 1500 to 2500 ms.

This study employed the same experimental paradigm as Min et al. [20,40] did. Pairs of colored figures randomly drawn from a set of red or green circles or squares were used as stimuli (cf. Fig. 3). The areas of circles and squares were matched. Stimuli were presented for 500 ms, on a computer monitor placed in front of the subject at a distance of one meter. Two colored figures (a stimulus set) were presented side-by-side on a light-gray background at an eccentricity of 3° (visual angle), and each colored figure in a stimulus set subtended a 4° visual angle. All types of stimuli appeared approximately at random, with equal probability. Each stimulus presentation was followed by variable ISIs ranging randomly between 1500 and 2500 ms. We instructed subjects to remain centrally fixated and to press a button with the index finger of one hand if the target feature of the two presented stimuli ('color' in the color task and 'shape' in the shape task) was the same across the two presented stimuli, and to press another button with the other hand if it was not. Subjects were asked to press the button as quickly as possible. To limit the experimental paradigm to top-down processing, we employed the same physical stimuli across both tasks while the subjects performed different tasks.

The experiment consisted of two task-sessions: a color task and a shape task. Stimuli in each task were presented in two blocks separated by short rest periods. Response hands and the order of tasks were counterbalanced across subjects. The experiment consisted of 384 trials for each task. Only trials with correct responses were further analyzed.

### EEG recording

EEG was recorded using a GRASS 15A54 amplifier (Grass Technologies, USA) with 21 sintered Au/Ag-electrodes. Their locations, in accordance with the international 10-20 system were as follows: AFz, Fp1, Fp2, Fz, F3, F4, F7, F8, Cz, C3, C4, T3, T4, Pz, P3, P4, T5, T6, Oz, O1 and O2. We also placed an electrode on each mastoid for the linked reference and a ground electrode at nasion. Eye movement activity was monitored with two additional electrodes placed supra-orbitally on both eyes and referenced to the linked mastoids. Electrode impedances were kept below 10 kΩ prior to data acquisition. EEG was sampled at 1000 Hz (analogue band-pass filter 0.1-100 Hz) and stored for off-line analysis. Data were epoched from 1000 ms prestimulus to 1000 ms poststimulus. Epochs containing eye-movements or other artifacts (maximum amplitude ± 70 μV or electrode drifts) were rejected (As a result, the average rejection rate was 25.6%).

### Wavelet transformation

The power of oscillatory activity was investigated by convolving the EEG signals with Morlet wavelets [52,53]. The Morlet-convolved signal shows a Gaussian envelope with a temporal standard deviation (σ_t_) and a spectral standard deviation (σ_f _= 1/(2πσ_t_)) around its central frequency (*f*_0_):(1)

To have unit energy at all scales, the wavelet functions should be normalized prior to the convolution. For the Morlet wavelet transform, the normalization parameter *A *is σ_t_^-1/2^π^-1/4^. A wavelet family is characterized by a constant ratio (*f*_0_/σ_f_), and we employed a wavelet family with *f *_0 _ranging from 1 to 30 Hz in 1 Hz steps (cf. Fig. 2) and 3.8 as its constant ratio in order to consider optimal spectral-temporal resolution of lower frequency bands such as theta and alpha activities [54].

The wavelet transform was performed for each individual trial, and the absolute values of the resulting transforms were averaged. This measure of signal amplitude in single trials reflects the total activity for a certain frequency range, irrespective of whether it is phase-locked to the stimulus or not. We will refer to this measure as the total activity, since it includes evoked as well as induced activity. In the present study, we computed the power (μV^2^) of oscillatory activity.

### Analytic methods

Since we were interested in EEG oscillatory activity around both anterior and posterior regions, we selected for further analyses the two regions of interest (ROIs): anterior ROI (including the electrodes AFz, F3, Fz and F4) and posterior ROI (including the electrodes Pz, O1, Oz and O2), which showed most pronounced responsiveness to prestimulus theta and alpha power, respectively (cf. Fig. 1). In the present study, we confined alpha activity to the frequency range from 8 to 13 Hz and theta activity to the frequency range from 4 to 7 Hz. For the prestimulus total power of these activities, we computed mean power in the time window from 300 to 50 ms prior to stimulus onset in each frequency range. This time window was chosen to avoid the temporal smearing of poststimulus activity into the baseline [52], while trying to take the time window as close to the stimulus onset as possible and also to include a reasonable period that included at least one cycle of both alpha and theta frequencies. No baseline correction was applied to the total power, since total alpha power in a prestimulus period would vanish after baseline correction. For the poststimulus total theta power, we measured maximum theta power in the time window from 200 to 450 ms poststimulus. This time window was determined on the basis of the grand averages (most pronounced and free of offset responses) and the previous studies about task-related (late) theta response were taken into account [54,55]. In the case of the poststimulus total alpha power, we chose the same time window, when the poststimulus amplitude modulation of alpha activity was most pronounced, and took minimum alpha power, since the grand-average of total alpha activity decreased after stimulation, as shown in Figs. 1 and 2. This phenomenon is typically referred to as 'alpha-blocking'.

Reaction times and accuracy of task-performance (error rates) were also measured for the behavioral analysis. Reaction times were collected within their individual 95% confidence intervals. These behavioral measures were analyzed with a repeated measures analysis of variance (ANOVA) that included a within-subjects factor labelled as 'task' ('color task' versus 'shape task'). In addition to this factor, we used a within-subjects factor labelled as 'ROI' ('anterior' versus 'posterior') to compare the electrophysiological activity between anterior and posterior brain areas.

## Authors' contributions

HJP has initiated this study. BKM has designed the experiment and drafted the manuscript. BKM and HJP carried out the recording of the data and ran the analyses and discussed the results. All authors have read and approved the final manuscript.
